# Simultaneous Radiological and Fiberendoscopic Evaluation of Swallowing (“SIRFES”) in Patients After Surgery of Oropharyngeal/Laryngeal Cancer and Postoperative Dysphagia

**DOI:** 10.1007/s00455-019-09979-8

**Published:** 2019-02-14

**Authors:** M. Scharitzer, I. Roesner, P. Pokieser, M. Weber, D. M. Denk-Linnert

**Affiliations:** 1 Department of Biomedical Imaging and Image-Guided Therapy, Medical University of Vienna, Waehringer Guertel 18-20, 1090 Vienna, Austria; 2 Division of Phoniatrics-Logopedics, Department of Otorhinolaryngology, Medical University of Vienna, Waehringer Guertel 18-20, 1090 Vienna, Austria; 3 Unified Patient Project, Medical University of Vienna, Waehringer Guertel 18-20, 1090 Vienna, Austria

**Keywords:** Cineradiography, Endoscopy, Deglutition, Deglutition disorders, Respiratory aspiration, Pharyngeal neoplasms

## Abstract

To compare the results of a simultaneously performed videofluoroscopic swallowing study and fiberendoscopic evaluation of swallowing in patients with dysphagia after surgery and radiotherapy for oropharyngeal or laryngeal cancer. This prospective study included 31 patients who were examined simultaneously with a standardized protocol. The fiberendoscopic and videofluoroscopic swallowing loops were independently scored by two otorhinolaryngologists/phoniatricians and two radiologists. The presence of penetration/aspiration, the amount of pharyngeal residues and the position of the bolus head when triggering of pharyngeal swallow begins were evaluated. Generalized linear models were used to model the impact of rater, method, bolus and quantities as well as specified moderation effects on scorings. In addition, post hoc Wilcoxon tests were used. Rater agreement was assessed using weighted kappas and their 95% confidence intervals. A total of 202 swallow sequences in 29 patients was evaluated. Interrater agreement was substantial to excellent for both methods (weighted *k* = 0.979–0.613). Significant differences between both methods were found when assessing the penetration-aspiration scale (*p *= 0.001, tendency of higher scores by videofluoroscopic (median = 2.59) as opposed to fiberendoscopic (median = 2.14) and the residue severity scores in the valleculae (*p *= 0.029) and the sinus piriformes (*p *= 0.002) with larger residues scored by fiberendoscopic evaluation of swallowing. No significant differences were found regarding the time point of triggering (*p *= 0.273). Simultaneous evaluation of swallowing with FEES and VFSS showed significantly different results in symptomatic patients after tumor operation and radiotherapy.

**Electronic supplementary material:**

The online version of this article (10.1007/s00455-019-09979-8) contains supplementary material, which is available to authorized users.

## Introduction

Swallowing disorders after surgery and concomitant radiotherapy for oropharyngeal and laryngeal cancer are found in up to 50–70% of patients, 20% of whom will need nutrition through a PEG tube [1, 2]. Often, symptoms have detrimental effects on the quality of life and health starting immediately after treatment and with variable onset and extent of improvement over time [3]. Swallowing impairment is due to restrictions in mobility and loss of sensitivity which can be due to both the resection of anatomical structures necessary for swallowing and the aftereffects of radiotherapy. It is mandatory to asses the risk of aspiration which can result in the most important complication of postsurgical dysphagia—a concomitant pneumonia. For a personalized dysphagia management and therapy it is also essential to assess the structural and functional deficits of swallowing, e.g. post swallow residue or triggering of the swallow reflex in relation to bolus passage. Patients’ subjective symptoms vary significantly from quantitative assessments of swallowing function [4], emphasizing the necessity for objective diagnostic examination tools.

Fiberendoscopic evaluation of swallowing (FEES) and videofluoroscopic swallowing studies (VFSS) are the established instrumental methods of choice for assessment of oropharyngeal dysphagia [5, 6]. Both types of studies have advantages and disadvantages and are applied in a complementary fashion. The major advantage of FEES, which visualizes the pharyngeal stage of swallowing, is the direct examination of structural alterations in the upper aerodigestive tract as well as the assessment of secretions. Neither can be evaluated by VFSS. Disadvantages include the phenomenon of “white out,” found during bolus passage during the swallow, in which an area is missing from endoscopic viewing [7]. Videofluoroscopy shows the bolus flow in relation to structural movements throughout the upper aerodigestive tract and allows an overall overview of the swallowing tract with the concurrent disadvantage of the need for ionizing radiation, as well as diminished assessment of structural alterations.

A direct correlation between the results of both methods is difficult because they are performed at different time points. Bolus size and consistencies and individual general conditions may vary and therefore result in different findings. Through a simultaneous examination, both the examination conditions, as well as the general condition of the patient, can be ruled out as a variable. To date, only a few studies have analyzed the simultaneous application of both diagnostic methods [6, 8–11]. These studies have focused on the examination of unimpaired swallowing in healthy subjects [6], or the assessment of the risk of aspiration [8], and pharyngeal residue [9, 10] in a small number of dysphagic patients without assignment to a specific clinical group. Coffey et al. evaluated simultaneously performed examinations in patients after laryngectomy [11]. Especially in patients after surgery in the area of the oropharynx and larynx and concomitant radiotherapy, the combination of structural and functional deficits represents a particular challenge for imaging modalities. In addition, the timepoint of aspiration in relation to bolus flow and triggering of the swallowing reflex have not been published as yet, to our knowledge.

Therefore, the aim of our study was to compare findings of simultaneously performed FEES and VFSS in a prospectively selected patient group by using a predefined examination protocol. The obtained information is intended to show the differences in evaluating swallowing pathologies between both methods and thus to help with the interpretation of divergent findings.

## Materials and Methods

The study was approved by the local ethics committee. All patients gave written, informed consent for study enrollment.

### Inclusion and Exclusion Criteria

Consecutive patients scheduled for assessment of dysphagia by FEES and VFSS to assess further diet modifications or the necessity of a change to or a continuation of nonoral feeding were included in this study. Inclusion criteria were as follows: (a) age of 18 years and older after operation for pharyngeal or laryngeal carcinoma; (b) ongoing or completed radiotherapy; (c) presence of postoperative dysphagia with high risk for aspiration; and (d) stable clinical condition. Exclusion criteria were as follows: (a) pregnancy; (b) contraindication to FEES, including hemophilia; inability to tolerate transnasal insertion of the flexible endoscope; (c) unstable or unresponsive patient; and (d) inability to ingest oral contrast medium.

### Simultaneous Radiological and Fiberendoscopic Evaluation of Swallowing: Protocol

The investigation was performed in the Department of Radiology. All procedures were carried out by an otorhinolaryngologist/phoniatrician (I.R., 15 years of experience) from the Division of Phoniatrics-Logopedics, Department of Otorhinolaryngology and a radiologist (M.S., 17 years of experience) from the Department of Radiology. Patients were investigated in the lateral standing position within the fluoroscopy unit (Siemens Sireskop, Erlangen, Germany, under-table X-ray tube). The transnasal insertion of a flexible endoscope (3.2 mm Fiber Rhinolaryngoscope Olympus ENF-GP) was performed by the otorhinolaryngologist. The tip of the endoscope was aimed to be placed at the upper rim of the epiglottis. Visibility of the endolaryngeal and pharyngeal structures was optimized by repositioning the endoscope during the FEES. An EndoCompact Mobile Unit (Xion, Berlin, Germany) for ENT with integrated LED illumination and compact camera was connected to this endoscope, enabling automatic storage of the endoscopic visual and audio data.

Videofluoroscopy was performed simultaneously with digital storage of high-resolution images (video matrix 1024 × 1024) and a frame rate of 25 images per second on our in-house PACS system (Agfa, Impax). The field of view included the upper border of the nasopharynx to the level below the pharyngoesophageal segment including the proximal esophagus. Radiation safety for examiners was ensured by using a movable tableside shielding device, protective aprons, and thyroid shields.

The contrast medium used was a water-soluble, non-ionic contrast medium (Iopamidol, Gastromiro^®^, Bracco, Austria), which was mixed with blue food dye for endoscopic examination. The study protocol is based on a model called the “Volume Viscosity Test” published by Clavé [12]. This clinical screening test uses boluses of different volumes and viscosities to identify clinical signs of impaired efficacy and safety of swallowing. It starts with nectar-like thickened contrast medium, and includes the application of liquid and pudding-like consistencies with different bolus volumes depending on the individual swallowing pattern. Bolus volumes were increased beginning with 3 ml to 5 ml, 10 ml and 20 ml at most. For preparation of different consistencies, a modified instant corn starch product (Resource, ThickenUp^®^, Nestlé, Swiss) was used as thickener. The proper consistency was controlled by using the International Dysphagia Diet Standardisation Initiative IDDSI [13]. Bolus volumes were placed into the patient’s mouth via spoon or cup.

If high degree of aspiration was seen by one or both techniques, the increase of bolus volume was stopped for the safety of the patient and the next consistency according to the study protocol was tested.

### Image Analysis

Evaluation was performed by two experts in otorhinolaryngology/phoniatrics (> 15 years of experience, respectively) assessing anonymized FEES loops and by two experts in radiology (> 17 years of experience, respectively), assessing anonymized VFSS loops. The VFSS and FEES video loops were cut into single swallow clips and presented in a randomized order and a blinded fashion with respect to the other imaging modality. Clinicians were blinded to the patient´s clinical history. Videos were evaluated without audio recording to reduce recall bias. Evaluation included four parameters: (1) presence of penetration or aspiration by means of the Penetration Aspiration-scale (PAS) [14], (2) time of aspiration in relation to swallowing phase (pre-, intra-, and postdeglutitive) in the presence of a PA-scale > 5, (3) amount of pharyngeal residues within the valleculae and the sinus piriformes using a three-part scale (none or mild residues, moderate and severe residues, the latter referring to > 50% filling), and (4) the start of the triggering of the swallowing reflex using a four-part scale (start of the swallowing reflex when contrast medium passed the (1) level of the mandibula/vallecular pit, (2) epiglottis, (3) piriform sinus, (4) no visible triggering).

### Statistical Analysis

All statistical calculations were performed using IBM SPSS Statistics for Windows version 24.0. An a priory sample size calculation revealed that 196 single swallows would be needed to obtain a power of 80% to detect the expected difference using a Wilcoxon Matched Pairs Signed Ranks test with an alpha level of 5% (two-sided).

Ordinal data are described using medians. In addition, scores were grouped and crosstabs were calculated. For nominal data, absolute frequencies and percentages were used. In order to take multiple swallows per patient into account, generalized linear models were used to model the impact of rater, method, bolus and quantities as well as the moderation effects of rater, bolus and quantities on differences between methods on different scores. In addition, post hoc Wilcoxon tests were used. Rater agreement was assessed using weighted Cohen’s kappas and their 95% confidence intervals (CI). A *p* value equal to or below 5% was considered to indicate significant results.

## Results

Between December 2016 and June 2017, 31 patients were enrolled in the study. Two patients had to be excluded, one because he could not tolerate the nasopharyngeal endoscope, and the other, because videofluoroscopic images could not be stored due to technical problems. Therefore, the final population comprised 29 patients. Table 1 describes pertinent patient characteristics. In total, 202 swallowing sequences could be assessed by simultaneous evaluation (range 1–12 swallows/patient, mean 7, respectively).

**Table 1:** Summary of patient characteristics

Characteristic	*n *= 29
Age	
Mean (years)	63.5
Range (years)	48 to 90
Sex	
M	24
F	5
Site of disease	
Nasopharynx	1
Oropharynx	20
Larynx	8
Time of radiotherapy [32]	
Ongoing or < 90 days after completion of radiotherapy	20
> 90 days after completion of radiotherapy	9
Chemotherapy	
Ongoing chemotherapy	9
Completion of chemotherapy	7
No chemotherapy	13
Nutrition	
Oral	16
Partial nonoral	3
Complete nonoral	10
Feeding tube	
Nasogastric tube	4
Percutaneous endoscopic gastrostomy	9
Tracheostomy	16

Data are numbers of subjects

Interrater agreement for VFSS was excellent for the assessment of PAS, the amount of residues in the valleculae and piriform sinus, and was substantial for location of swallow trigger. Interrater agreement for FEES was excellent for the assessment of PAS and location of swallow trigger, and substantial for the amount of residues in the valleculae and piriform sinus (Table 2).

**Table 2:** Interrater agreement for variables assessed by VFSS and FEES

Modality	Weighted kappa	95% CI
VFSS		
PA-score	0.979	0.963–0.994
Retentions valleculae	0.819	0.748–0.890
Retentions piriform sinus	0.857	0.784–0.930
Time of triggering	0.771	0.689–0.853
FEES		
PA-score	0.911	0.864–0.959
Retentions valleculae	0.613	0.528–0.697
Retentions piriform sinus	0.762	0.686–0.837
Time of triggerung	0.828	0.750–0.906

*PA score* penetration aspiration score, *CI* confidence interval

**Fig. 1:**
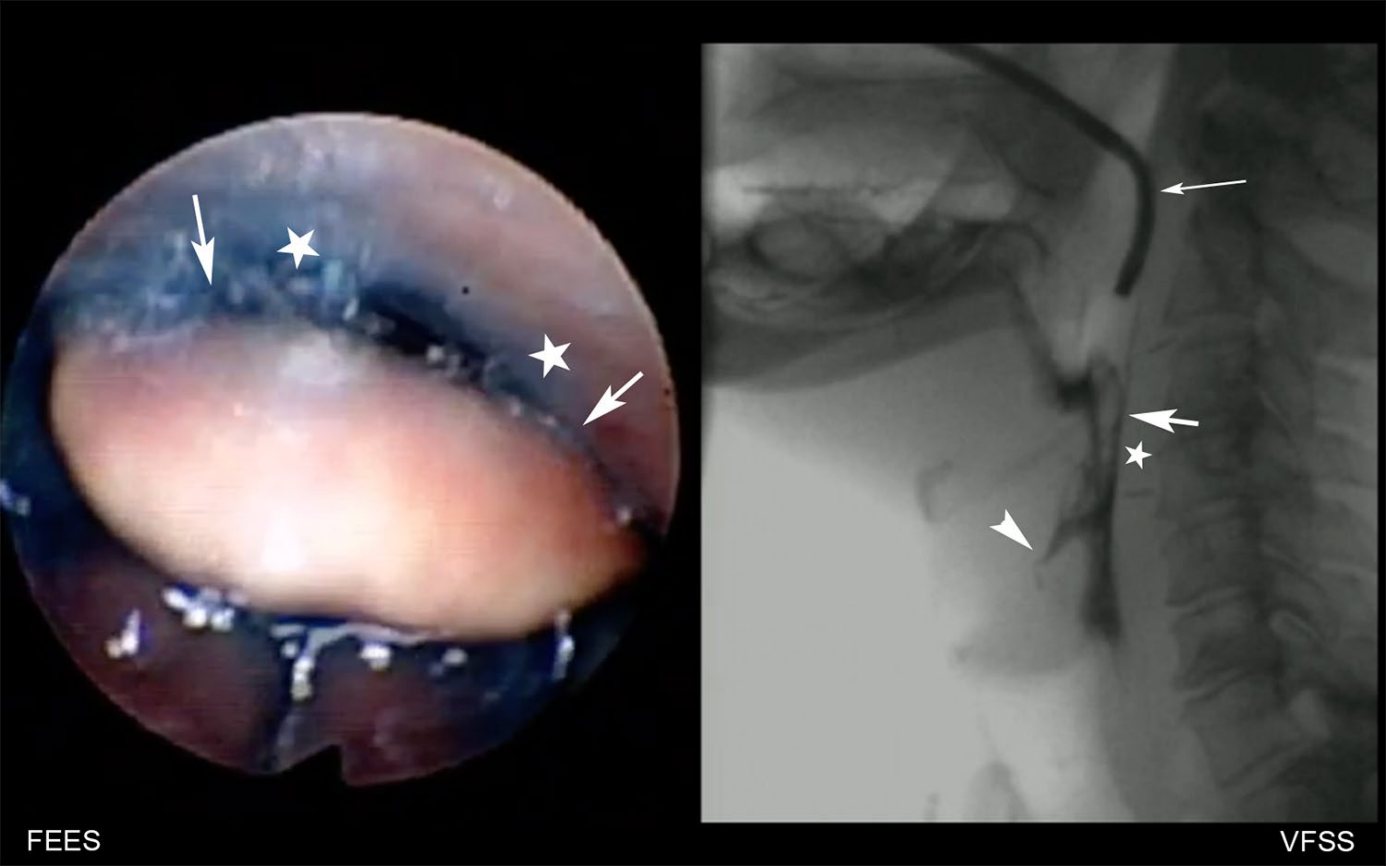
Simultaneous evaluation of FEES (left) and VFSS (right; arrow indicating endoscope). In this patient, the epiglottis (short arrow) is not tilting during swallowing 5 ml of nectar consistency and has direct contact to the dorsal pharyngeal wall (asterisks), making a direct endoscopic view into the laryngeal vestibule impossible and resulting in a missing diagnosis of intradeglutitive penetration as seen during VFSS (arrowhead)

**Fig. 2:**
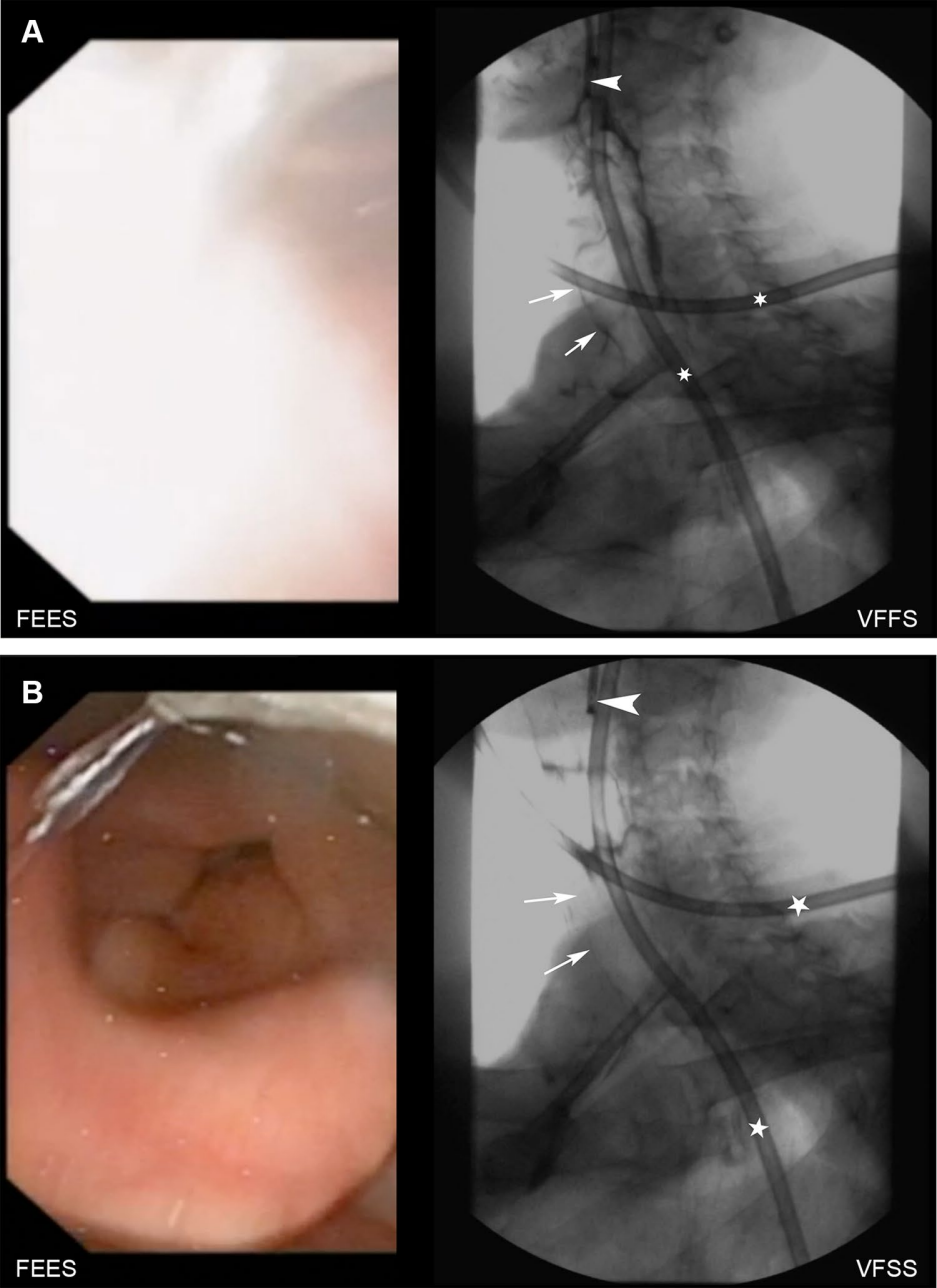
**a** shows the swallow of a 5 ml bolus of liquid consistency. On the left, an intradeglutitive “white-out” is seen on FEES, VFSS shows intradeglutitive silent aspiration (arrows; arrowhead: endoscope, asterisks: nasogastric tube). **b** shows same patient after swallowing: no intralaryngeal or intratracheal contrast medium is seen on FEES as well as on VFSS (arrows, arrowhead: endoscope, asterisks: nasogastric tube)

Significant differences between both modalities were found when assessing the penetration-aspiration scale by FEES and VFSS (*p *= 0.001, tendency of higher scores by VFSS (median of median scores of both raters = 2.59) to FEES (median of median scores of both raters = 2.14), the difference depending on raters (*p *= 0.016) and consistency (*p *= 0.039) (Fig. 1; Online Resource 1). Penetration aspiration scale showed also significant differences between different amounts of contrast material (*p *= 0.008). When grouping the PAS into normal swallow (PAS = 1), penetration (PAS = 2–5) and aspiration (PAS = 6–8), the differences between penetration-aspiration scale between both modalities were also significant (*p *= 0.002), depending on rater (*p *= 0.045) and consistency (*p *= 0.027). Detailed results are shown in Table 3. In 16/202 swallows (8% of all swallows) of ten patients (35%), VFSS detected aspiration (PAS > 5), that was not detected by FEES (Fig. 2; Online Resource 2) In four of these patients, FEES scored deep penetration (PAS = 5 in three patients and PAS = 4 in one patient), high penetration in two and no visible penetration in three patients. In one patient with a PAS of 5 scored by FEES and 8 by VFSS during swallows of nectar and liquid consistency, a single swallow of pudding revealed a PAS of 8 by both modalities. Of these ten patients, six patients had intradeglutitive and four had postdeglutitive aspiration during VFSS. In two swallows of one patient, FEES assessed a PAS of 6 (intradeglutitive penetration, that led to consecutive aspiration), which was scored as deep penetration with residues (PAS = 5) by VFSS. In all swallows with assessment of aspiration by VFSS and FEES (*n *= 15), classification of time of triggering was completely consistent with both methods (predeglutitive *n *= 2, intradeglutititve *n *= 7, postdeglutitive *n *= 6).

**Table 3:** Penetration-aspiration scores for VFSS and FEES and different bolus volumes and consistencies

Grouped PA-score	VFSS			
	1	2–5	6–8	Total
FEES				
3 m1				
1	39	6	3	48
2–5	1	12	5	18
6–8	0	1	8	9
Total	40	19	16	75
* p*-value	0.003			
5 ml				
1	34	4	1	39
2–5	1	10	3	14
6–8	0	1	5	6
Total	35	15	9	59
* p*-value	0.052			
10 ml				
1	27	5	1	33
2–5	0	2	2	4
6–8	0	0	2	2
Total	27	7	5	39
* p*-value	0.007			
20 ml				
1	20	6	1	27
2–5	0	2	0	2
Total	20	8	1	29
* p*-value	0.011			
Nectar				
1	51	9	3	63
2–5	1	5	5	11
6–8	0	0	3	3
Total	52	14	11	77
* p*-value	< 0.001			
Pudding				
1	40	2	1	43
2–5	0	13	0	13
6–8	0	1	9	10
Total	40	16	10	66
* p*-value	0.257			
Liquid				
1	29	10	2	41
2–5	1	8	5	14
6–8	0	1	3	4
Total	30	19	10	59
* p*-value	0.001			

Chart shows the PA scores grouped in normal swallow, penetration and aspiration for both modalities for different bolus volumes and bolus consistencies

*PA score* penetration aspiration score

**Fig. 3:**
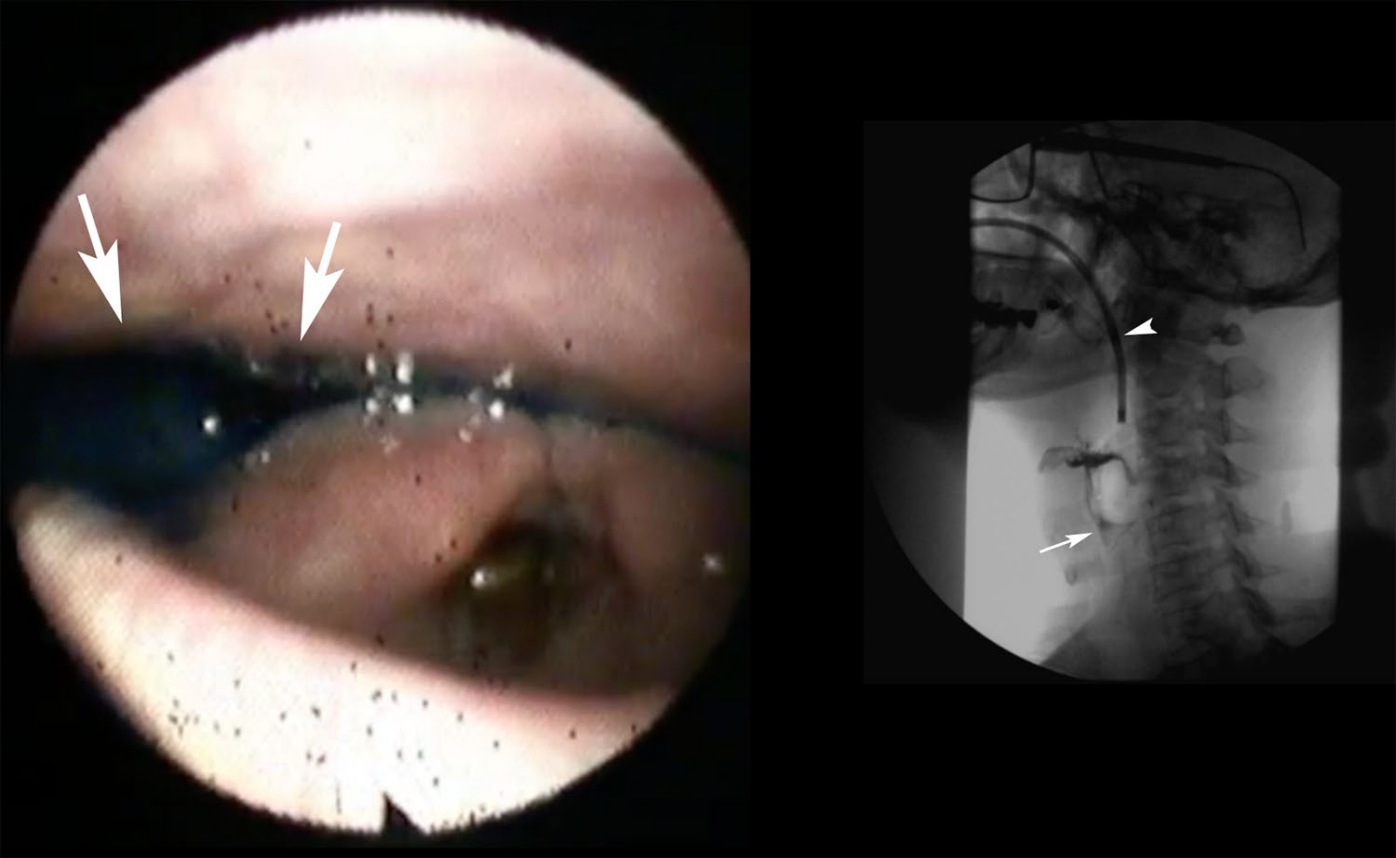
Left image shows FEES of a patient with severe pooling of saliva and secretions as well as contrast medium after swallowing in the piriform sinus (arrows) whereas VFSS shows only mild residues of contrast medium within the piriform sinus (arrow; arrowhead: endoscope)

Significant differences between both methods were found when assessing the residue severity scores in the valleculae (*p *= 0.029), depending on rater (*p *< 0.001) with larger residues scored by FEES (median of median scores of both raters = 1.67) compared to VFSS (median of median scores of both raters = 1.52). Residue severity scores in the piriform sinus were also significantly different (*p *= 0.002) with larger residues scored by FEES (median of median scores of both raters = 1.51) compared to VFSS (median of median scores of both raters = 1.32) as well (Fig. 3; Online Resource 3).

**Fig. 4:**
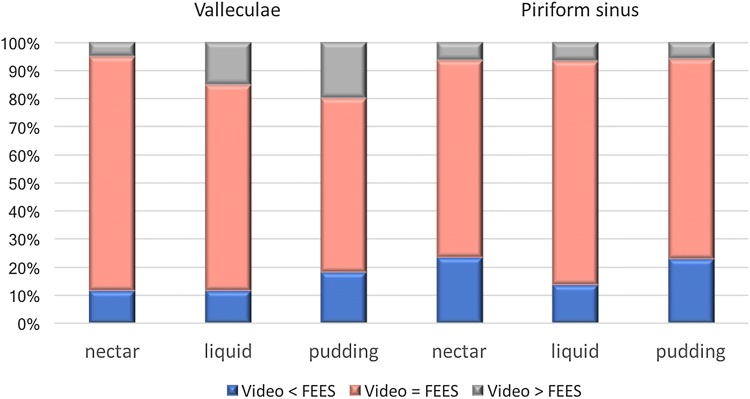
Different evaluation results of pharyngeal residues in the valleculae and the piriform sinus between VFSS and FEES of the first rater each at the entire number of evaluations per consistency and localization

Regarding the bolus consistency of retained contrast medium, differences between both modalities were significant for nectar (*p *= 0.005) and pudding (*p *= 0.011) consistencies, but not for liquid consistencies (*p *= 0.153), (Fig. 4). Time of triggering could be assessed in 193/202 swallows by FEES. Compared to VFSS, statistical analysis showed no significant differences in the scoring time for triggering (*p *= 0.273), with a later onset of swallowing assessed by VFSS (median = 1.63) compared to FESS (median = 1.39).

## Discussion

In our study, we demonstrated that VFSS and FEES may show different results regarding the evaluation of oropharyngeal swallowing in patients after surgery and concomitant radiotherapy for pharyngeal or laryngeal cancer. Statistical significant differences were observed for the assessement of the penetration/aspiration scale and residue severity scores in the piriform sinus, while showing overall good to excellent interrater agreement for both modalities. Especially in patients with head and neck-cancer, the preservation of swallowing function has been rated as important as oncologic treatment [15].

Radiotherapy reduces the quality of life in patients with head and neck cancer in addition to surgical defects with reported incidences of silent aspiration and lack of subsequent cough reflex of 22–67% [16] and a risk of malnutrition and aspiration, estimated to be 36–94% in this particular patient population [17]. Development of mucositis, edema of soft tissues, xerostomia, and tissue swelling lead to acute dysphagia, whereas late tissue responses such as fibrotic reactions, damage to the vasculature and atrophy may cause late onset of dysphagia [16]. Pneumonia is found in up to one in four patients with head and neck cancer and concurrent chemoradiotherapy [18]. Nguyen presented a study of modified barium swallow examinations in 63 patients before and after chemoradiation for head and neck cancer and found aspiration in 59%, 9% of which had a fatal outcome [19].

Videofluoroscopy and FEES continue to be the most widely used dynamic imaging methods for the evaluation of swallowing physiology and rehabilitation planning of patients with dysphagia. It has been reported, that each modality yields false-negative and false-positive results and much controversy exists about which method should be preferred. A number of studies have been published to evaluate the relative benefits of each investigation method from a variety of perspectives, both in adults [20–22] and in children [23]. The first study that compared FEES and VFSS was published in 1991, as reported by Langmore et al. [24]. When comparing both methods, most studies were performed consecutively, thus posing a risk of intersubject and intrasubject variability.

Importantly, only very few studies report on the simultaneous comparison of swallowing with FEES and VFSS. Logemann examined eight young adult males comparing two different endoscopic positions in relation to videofluoroscopy [6]. Pisegna performed simultaneous investigation in two patients and 55 clinicians evaluated two swallows [10]. Kelly compared videofluoroscopy and FEES simultaneously with regard to the assessment of penetration and aspiration and pharyngeal residue [8, 9]. Recently, a British group investigated simultaneous FEES and VFSS in patients after laryngectomy [11]. In most studies, patients were suffering from dysphagia of mixed etiology.

Overall, study results were comparable to our results, which showed higher residue scores with FEES than with videofluoroscopy. However, penetration and aspiration were perceived as more severe on FEES than on videofluoroscopy in a study by Kelly, which is in contrast to our results. In our study, the fiberendoscopic view was significantly diminished in many swallows. This was due to severe mucosal edema of the supraglottic structures with limited visualization of the endolarynx. Severe secretions and bolus residue additionally blurred the vision when caught on the tip of the endoscope, representing a major challenge in this patient group. As opposed to our patient population, Kelly excluded nonoral patients and patients with high risk of aspiration in her study group of 15 dysphagic patients with varying clinical diagnoses. In her study, the time point of aspiration related to swallowing was not further subclassified.

In our patients with missed aspiration by FEES, VFSS could detect intradeglutitive aspiration in six and postdeglutitive aspiration in four patients. This may be due to the fact, that patients, after radiation of neck cancer, often present with a swollen epiglottis and incomplete laryngeal closure, which leads to intradeglutitive aspiration and diminished visual insight into the laryngeal vestibule by FEES. However, in one patient, aspiration was detected only by FEES, due to the longer examination protocol and the visualization of the entry of contrast medium below the vocal folds when VFSS had already stopped fluoroscopy in the attempt to keep radiation exposure on a minimal level. In addition, in 40% (*n *= 13 out of 33 swallows with aspiration assessed by at least one of both modalities) of swallows, aspiration occurred postdeglutitively and in all of those incidences, moderate or severe pharyngeal residues were present. This matches the observations of other groups, that especially in patients with head and neck cancer, aspiration often occurs after the swallow due to residue spilling into the laryngeal vestibule as it opens [25].

We used different volumes and consistencies of contrast medium, although few comparative studies between FEES and VFSS exist, that address this fact. Although a 5 ml thin liquid bolus has been reported as commonly used in VFSS protocols in head and neck cancer patients [26], we started with a 3 ml bolus, since a significant group of our patients was non-oral prior to the examination. Our separate analysis of bolus volumes and consistencies also showed significant differences for aspiration detection for liquid and nectar consistency, as well as for the classification of residues in the piriform sinus, except for large volumes and liquid consistencies.

Interpreting swallowing sequences by FEES and VFSS are mostly based on visual judgement, which poses the risk of subjective erroneous assessment. There exist visuoperceptual scales for assessing pharyngeal residues, that have been published for both methods [27, 28]. For assessment of timepoint of initiation of pharyngeal swallow in relation to bolus flow Bonnie Martin-Harris has validated a 4-point scale [29]. We combined scale 0 and 1 (bolus head at posterior angel of ramus and bolus head at vallecular pit), since in older patients both timepoints can be considered to be within normal limits, as stated by Stephen et al. [30]. Contrary to former studies [11, 31–33], our results showed good interrater variability for both modalities. This may be due to the fact that our readers were highly qualified, which is reported to increase consistency between raters´ results [32, 33]. Only the assessments of residues within the valleculae between both FEES raters was lower, with a constant tendency of higher scores by rater 2, showing an individual different estimation of degree of residues despite the use of a standardized, clearly defined residue severity scale. Good interrater reliability may have been influenced by a smaller number of raters compared to other studies.

Our goal was not to look for a gold standard, but to show that both methods have their specific advantages and limitations and are more sensitive for particular aspects of swallowing assessment. As Kelly stated, neither tool alone leads to the correct answer with regard to the penetration aspiration scale [8]. Aviv and Murry also reported, that neither FEES nor VFSS alone allow the clinician to safely make a decision to feed the patient [20].

In order to compare both modalities, only variables presumably assessable in both of them could be evaluated in this study. Therefore, the significant advantages of the individual methods could not be taken into account. In addition, our selected patient group accounts for the most severely limited dysphagic patients with large surgical defects and mucosal alterations after radiotherapy contributing to the impairment. Consequently, massive swelling as found in patients with ongoing or recently completed radiotherapy may limit the examination accuracy for endoscopy and therefore makes videofluoroscopy more preferable for assessing aspiration in this patient group. On the other hand, distinctive residues of saliva and secretions cannot be assessed by VFSS and lead to a more severe scoring of residues within the piriform sinuses by FEES. Another possible explanation for different residue ratings may be the fact, that severe swelling of valleculae and piriform sinus with residues on top may give the impression of high grade residues during endoscopy. A possible limitation was the heterogeneous group of participants in regard to tumour site, time since radiotherapy, nutritional status and need for a feeding tube. 55% of our patients had a tracheostomy tube, a condition associated with oropharyngeal dysphagia. Additionally, video sequences were evaluated without sound recording to reduce bias by spoken words during the investigation, such as the request to cough after a deep aspiration. Nevertheless, in six out of ten patients with missed aspiration during FEES, the PA score assessed by VFSS was 8 due to absent signs of coughing after aspiration of contrast medium.

In conclusion, our study shows that videofluoroscopy adds significant and crucial information to the findings of the FEES for this specific patient group, namely the detection of aspiration and the quantity of pharyngeal residues. Both modalities should not be considered as interchangeable procedures, and, with regard to the relative benefits of each procedure, both provide relevant information in dysphagic patients after pharyngeal or laryngeal cancer and radiotherapy. As complementary instrumental examinations, they can be performed simultaneously in a multidisciplinary approach.

## Electronic supplementary material

Below is the link to the electronic supplementary material.

Supplementary material 1 (MP4 9071 kb). Electronic Supplementary Material 1. Video that demonstrates a patient with an absent tilting of the epiglottis resulting in lack of a direct endoscopic view into the laryngeal vestibule by FEES and missing diagnosis of intradeglutitive penetration as seen during VFSS. mp4

Supplementary material 2 (MP4 7081 kb). Electronic Supplementary Material 2. Video that demonstrates a “white-out” of FEES prohibiting direct visualization of aspiration during the swallow as seen on VFSS. After the swallow, no intralaryngeal or intratracheal contrast medium is seen on FEES as well as on VFSS. mp4

Supplementary material 3 (MP4 6714 kb). Electronic Supplementary Material 3. Video that demonstrates severe pooling of saliva mixed with residues of contrast medium in the piriform sinus seen on FEES after swallowing and only mild residues of contrast medium seen on VFSS. mp4

## Abbreviations

VFSS: Videofluoroscopic swallowing study
FEES: Fiberendoscopic evaluation of swallowing
PAS: Penetration aspiration scale
PEG: Percutaneous endoscopic gastrostomy
